# Supplementary material for: Building a community to engineer synthetic cells and organelles from the bottom-up
Source: eLife. 2021 Dec 20;10:e73556. doi: 10.7554/eLife.73556 (PMC8716100; doi:10.7554/eLife.73556)

**Building a community to engineer synthetic cells and organelles from the bottom-up (Staufer *et al*. 2021 *eLife* 10:e73556)**

**Supplementary file 1**

**Table S1.** Presentations at SynCell2020.

**Table S2.** Presentations at the SynCell2021 Spring Lecture Series with Build-A-Cell.

**Table S3.** Featured and Contributed Oral Presentations at SynCell2021.

**Table S4.** Poster Presentations at SynCell2021.

**Table S5.** Lightning Talks at SynCell2021. (Presenters chosen by jury from poster presenters.)

**Box S1.** Quotations from the early-career panelists of the SynCell20/21 workshop.

**Box S2.** Quotations from the early-career panelists of the SynCell20/21 workshop on the future challenges in the field.

**Table S1.** Presentations at SynCell2020.

| **Presenter** | **Type** | **Title** | **Date** | **Youtube Link** |
| --- | --- | --- | --- | --- |
| Reinhard Lipowsky | Tutorial | Understanding and controlling the morphological complexity of biomembranes | 5/26/2020 | <https://www.youtube.com/watch?v=V5kHvKBevrM> |
| Karin Jacobs | Tutorial | Unspecific interactions in or of cell-like compartments: theory & experiments | 5/26/2020 |  |
| Michael Grunze | Program Highlight | Max Planck Matter-to-Life School | 5/26/2020 |  |
| Joachim Spatz | Keynote | Bottom-up assembly of a cell | 5/26/2020 |  |
| Darko Stefanovic | Tutorial | Development and application of DNA in nanoscale robotics | 5/27/2020 | <https://www.youtube.com/watch?v=FM-70enAeFo> |
| Jim Werner | Tutorial | Methods to visualize 3D dynamics | 5/27/2020 |  |
| Marileen Dogterom | Program Highlight | BaSyC | 5/27/2020 |  |
| Marileen Dogterom | Keynote | Building a functional cytoskeleton in synthetic cells | 5/27/2020 |  |
| Drew Endy | Keynote | Upwelling — Challenges arising from the bottom of Earth’s life well | 5/28/2020 | <https://www.youtube.com/watch?v=SyqalYP-bFg> |
| Laura De Laporte | Tutorial | Synthetic building blocks to grow functional tissues | 5/28/2020 |  |
| Nick Carroll | Tutorial | Liquid/liquid phase separation of intrinsically disordered proteins | 5/28/2020 |  |
| Kate Adamala | Program Highlight | Build-a-Cell | 5/28/2020 |  |
| Andrew Ellington Kate Adamala Eberhard Bodenschatz Michael Grunze | Panel Discussion | Future synthetic cell technologies for mitigation of viral pandemics | 5/28/2020 |  |

**Table S2.** Presentations at the SynCell2021 Spring Lecture Series with Build-A-Cell.

| **Presenter** | **Title** | **Date** | **Youtube Link** |
| --- | --- | --- | --- |
| Michael Jewett | Cell-free systems for synthetic cells, on-demand biomanufacturing, molecular sensing, and education | 3/15/2021 | Not posted. |
| Judee Sharon | DNA-mediated inducible liposome fusion | 3/15/2021 | <https://www.youtube.com/watch?v=VmGcd-Dn4gs&t=1s> |
| Ramin Golestanian | How living matter self-organizes while breaking action-reaction symmetry | 3/22/2021 | <https://www.youtube.com/watch?v=djrwu4tL8go&t=1207s> |
| Jan Steinkühler | Controlled division of cell-sized vesicles by low densities of membrane-bound proteins | 3/22/2021 | <https://www.youtube.com/watch?v=eq_aojgf-Vo&t=1097s> |
| Clyde Hutchison | Essential universal tasks for a minimal living cell | 3/29/2021 | <https://www.youtube.com/watch?v=8cCj-SuMM5o&t=2156s> |
| Oskar Stauffer | Bottom-up assembly of functional fully-synthetic extracellular vesicles | 3/29/2021 | <https://www.youtube.com/watch?v=wSHczi0RKwM> |
| Eberhard Bodenschatz | Synthonems: building synthetic cilia from the bottom up | 4/5/2021 | <https://www.youtube.com/watch?v=DknSmix52Ck&t=133s> |
| Lado Otrin | Artificial mitochondrion, bottom-up! | 4/5/2021 | <https://www.youtube.com/watch?v=-RZdhF_X2W0> |
| Felipe Garcia Quiroz | Membraneless and stimuli-responsive organelles | 4/12/2021 | <https://www.youtube.com/watch?v=TPF9V20YW7c&t=1305s> |
| Cesar Rodriguez-Emmenegger | Superselectivity in synthetic protocells | 4/12/2021 | <https://www.youtube.com/watch?v=TPX0-c1Dxbc> |
| Phillipe Bastiaens | A synthetic morphogenic perceptory system | 4/19/2021 | <https://www.youtube.com/watch?v=qu6Akbg7FDM&t=455s> |
| Zachary Manzer | Creating Biomimetic Interfaces Using the Cell-Free Synthesis of Transmembrane Proteins | 4/19/2021 | <https://www.youtube.com/watch?v=GV5Ko-giT98> |
| Andrew Ellington | Cell free operating systems for diagnostics | 4/26/2021 | <https://www.youtube.com/watch?v=itZFfBC9Ihc&t=1575s> |
| Amelie Benk | Controlled linkage of different proteins in Synthetic Cells | 4/26/2021 | Not posted. |
| Hendrik Dietz | Designing biomolecular devices and machines | 5/4/2021 | <https://www.youtube.com/watch?v=g3FDadvwbAE> |
| Kevin Jahnke | Proton gradients from light-harvesting E. coli control DNA assemblies for synthetic cells | 5/4/2021 | <https://www.youtube.com/watch?v=s5ITxtjx9Fc> |
| Neal Devaraj | Lipid sponge droplets as programmable synthetic organelles | 5/10/2021 | <https://www.youtube.com/watch?v=9eBRysSAWXQ> |
| Emiliano Altamura | Light-driven ATP production promotes mRNA biosynthesis inside hybrid multi-compartment artificial protocells | 5/10/2021 | <https://www.youtube.com/watch?v=P__aO639YJY> |
| Joachim Spatz | Mechanism in collective organizations of living and synthetic cells | 5/17/2021 | Not posted. |
| Ilia Platzman | Microfluidic approaches towards reconstitution of synthetic cells motility | 5/17/2021 | Not posted. |

**Table S3.** Featured and Contributed Oral Presentations at SynCell2021.

| **Presenter** | **Type** | **Title** | **Date** | **Youtube Link** |
| --- | --- | --- | --- | --- |
| Neha Kamat | Featured | Membranes matter: designing bilayer membranes to control functions of artificial cells | 5/19/2021 | <https://www.youtube.com/watch?v=h5abE8fBDRA&t=3467s> |
| Jacqueline De Lora | Contributed | Rational design for assembly of biologically inspired compartments | 5/19/2021 |  |
| Kerstin Gopfrich | Featured | A shortcut towards synthetic cell division | 5/19/2021 |  |
| Matthew Good | Contributed | Engineered synthetic membraneless organelles built from self-assembling disordered proteins to regulate cellular function | 5/19/2021 |  |
| Matt Lakin | Featured | Information processing in synthetic cells | 5/19/2021 |  |
| Alisina Bazrafshan | Contributed | Programmable DNA origami motors | 5/19/2021 |  |
| Kate Adamala | Featured | Lineage agnostic biology | 5/20/2021 | <https://www.youtube.com/watch?v=tsez_iAzJ5E> |
| Michael Levy | Contributed | Cell-free autonomous biogenesis of a ribosomal subunit | 5/20/2021 |  |
| James Carothers | Featured | Synthetic cell systems for scalable bio-production of plant natural products | 5/20/2021 |  |
| Eleonora Bailoni | Contributed | A multi-chamber flow dialysis setup for energy homeostasis in synthetic cells | 5/20/2021 |  |
| Tobias Erb | Featured | Fixing CO2 fixation: Building an artificial chloroplast drop by drop | 5/20/2021 |  |
| Roseanna Zia | Contributed | Cellular Stokesian dynamics”: a computational model for biological cells | 5/20/2021 |  |

**Table S4.** Poster Presentations at SynCell2021.

| **Presenter** | **Type** | **Title** | **Date** | **Youtube Link** |
| --- | --- | --- | --- | --- |
| Alessandra Griffo | Poster | Insights on the effect of cholesterol and sphingomyelin on tension and elasticity of plasma-like freestanding model membranes from natural lipids | 05/18/21 - 05/19/21 | https://www.youtube.com/watch?v=VnCqOzcBCSA |
| Jimin Guo | Poster | Biomimetic rebuilding of multifunctional red blood cells | 05/18/21 - 05/19/21 | https://www.youtube.com/watch?v=_-rhtpkVwHE |
| Paola Albanese | Poster | Single compartment approach for photo-autotrophic protocell preparation | 05/18/21 - 05/19/21 | https://www.youtube.com/watch?v=U_TaK9Z2WfI |
| Roberto Javier Brea Fernandez | Poster | Chemoselective generation of dynamic synthetic cells | 05/18/21 - 05/19/21 | https://www.youtube.com/watch?v=-zMyvdkDSaM |
| Ahanjit Bhattacharya | Poster | Lipid Sponge Droplets as Programmable Synthetic Organelles | 05/18/21 - 05/19/21 | https://www.youtube.com/watch?v=XeXkc-C6wNY |
| Telmo Diez Perez | Poster | Recombinant Intrinsically Disordered Proteins for Triggered Sequestration of Nucleic Acids via Liquid-Liquid Phase Separation | 05/18/21 - 05/19/21 | https://www.youtube.com/watch?v=pa-iR9_5KpE |
| Nika Marušič | Poster | Charge vs. SNARE-mediated fusion of biomimetic polymer/lipid hybrid compartments: Which one is more efficient? | 05/18/21 - 05/19/21 | https://www.youtube.com/watch?v=s0cSmIih6SA |
| Sadaf Pashapour | Poster | Generation of Extracellular Matrix Protein-based Microcapsules for Investigating Single Cells | 05/18/21 - 05/19/21 | https://www.youtube.com/watch?v=XJoB_rkwJtM |
| Tobias Neckernuss | Poster | Monitor, categorize and manipulate label-free water-in-oil droplets in microfluidic systems | 05/18/21 - 05/19/21 | https://www.youtube.com/watch?v=ggcjzq2yZyg |
| James Hindley | Poster | Building mechanosensitive signalling pathways in synthetic cells using membrane engineering | 05/18/21 - 05/19/21 | https://www.youtube.com/watch?v=9SxuHz9WfCk |
| Jefferson Smith | Poster | Light-activated gene expression in synthetic cells | 05/18/21 - 05/19/21 | https://www.youtube.com/watch?v=Lzx-Zl-8tC8 |
| Michele Partipilo | Poster | A minimal pathway for the regeneration of redox cofactors | 05/18/21 - 05/19/21 | https://www.youtube.com/watch?v=sBBj06mzJFo |
| Qi Wang | Poster | Influence of Breast Cancer Lipid Changes on Membrane Oxygen Permeability | 05/18/21 - 05/19/21 | https://www.youtube.com/watch?v=7GMO2YN_hcc |
| Marco de Oliveira | Poster | Genetic circuits based on serine-integrases as regulatory networks in the minimal cell Mycoplasma mycoides JCVI-Syn3A | 05/18/21 - 05/19/21 | https://www.youtube.com/watch?v=-stjWC7v-iQ |
| Satyam Khanal | Poster | Chemoenzymatic Generation of Phospholipid Membranes Mediated by Type I Fatty Acid Synthase | 05/18/21 - 05/19/21 | https://www.youtube.com/watch?v=a6BKQYCiHXo&feature=youtu.be |
| Hendrik Hähl | Poster | Pure protein bilayers and vesicles made from fungal hydrophobins: an alternative platform for synthetic cells | 05/18/21 - 05/19/21 | https://www.youtube.com/watch?v=2meKpdRZ6SE |
| Sebastian Restrepo-Cruz | Poster | Tetraspanin Scaffold Regulation of Epidermal Growth Factor Receptor Biology on the Plasma Membrane | 05/18/21 - 05/19/21 | Not posted. |
| Franky Djutanta | Poster | Hydrodynamically-active oily ocean surface as a cradle for the emergence of life | 05/18/21 - 05/19/21 | https://www.youtube.com/watch?v=TIC4bfBLBsY |
| Mark Walker | Poster | Engineering High Throughput Biosynthesis of Natural Product-Like Cyclic Peptides | 05/18/21 - 05/19/21 | https://www.youtube.com/watch?v=p-X8LUZjYC8 |

**Table S5.** Lightning Talks at SynCell2021. (Presenters chosen by jury from poster presenters.)

| **Presenter** | **Type** | **Title** | **Date** | **Youtube Link** |
| --- | --- | --- | --- | --- |
| Jefferson M. Smith | Lightning Talks | Light-activated gene expression in synthetic cells | 5/20/2021 | <https://www.youtube.com/watch?v=vuwiYb5bUEE> |
| Telmo Díez Pérez | Lightning Talks | Recombinant Intrinsically Disordered Proteins for Triggered Sequestration of Nucleic Acids via Liquid-Liquid Phase Separation | 5/20/2021 |  |
| Sadaf Pashapour | Lightning Talks | Generation of Extracellular Matrix Protein-based Microcapsules for Investigating Single Cells | 5/20/2021 |  |
| Hendrik Hähl | Lightning Talks | Pure protein bilayers and vesicles made from fungal hydrophobins: an alternative platform for synthetic cells | 5/20/2021 |  |

**Box S1.** Quotations from the early-career panelists of the SynCell20/21 workshop.


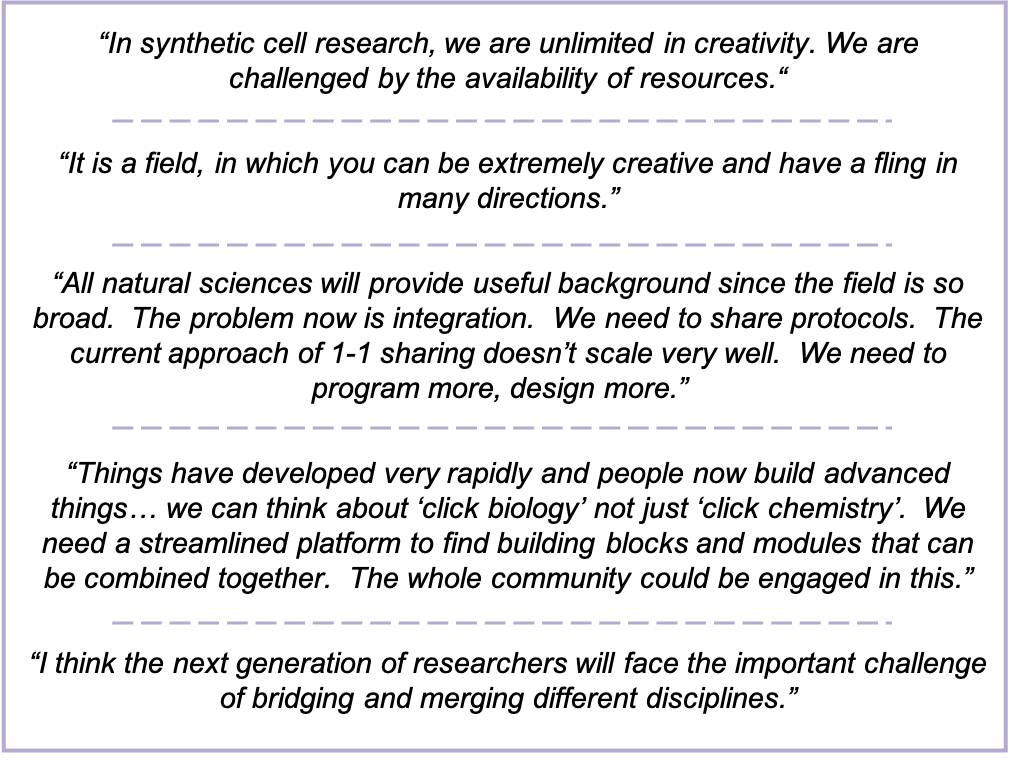


**Box S2.** Quotations from the early-career panelists of the SynCell20/21 workshop on the future challenges in the field.


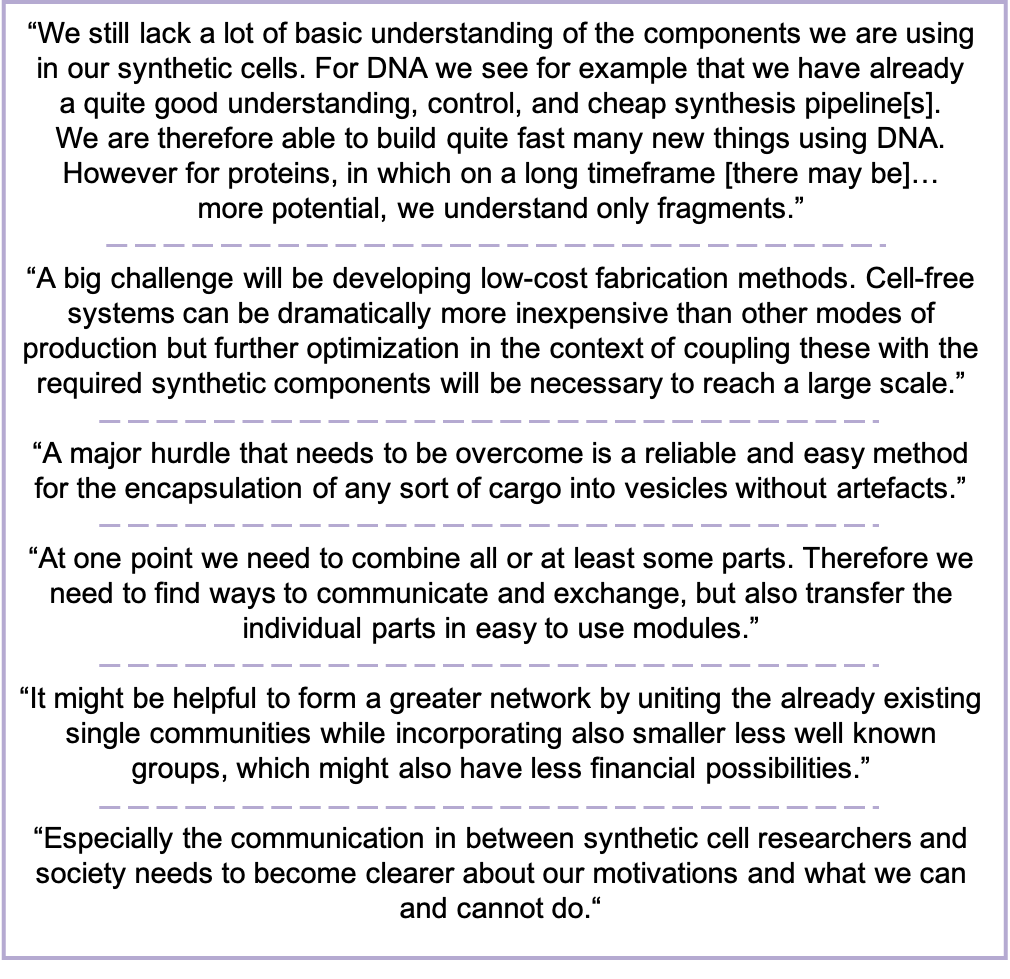

Supplement: Supplementary file 1. — Table S1: Presentations at SynCell2020. Table S2: Presentations at the SynCell2021 Spring Lecture Series with Build-A-Cell. Table S3: Featured and Contributed Oral Presentations at SynCell2021. Table S4: Poster Presentations at SynCell2021. Table S5: Lightning Talks at SynCell2021 (Presenters chosen by jury from poster presenters). Box S1: Quotations from the early-career panelists of the SynCell20/21 workshop on the future challenges in the field. [file elife-73556-supp1.docx]
